# Actinic cheilitis and associated factors in fishermen living in a rural riverside community in the Amazon

**DOI:** 10.1590/0103-644020256302

**Published:** 2025-07-11

**Authors:** Romyne Bastos Solano e Silva, Ana Paula Corrêa de Queiroz Herkrath, Matheus Albuquerque do Valle, Carla Rilane Bernardes Guimarães, Fernando José Herkrath, Juliana Vianna Pereira

**Affiliations:** 1 School of Dentistry, Federal University of Amazonas, Manaus, Amazonas, Brazil; 2 Fundação Oswaldo Cruz, Instituto Leônidas e Maria Deane, Manaus, Amazonas, Brazil

**Keywords:** Actinic cheilitis, rural population, occupational exposure

## Abstract

The study evaluated the prevalence of actinic cheilitis and associated factors in fishermen living in a rural riverside community in the Amazon, Brazil. A cross-sectional study was conducted with male artisanal fishermen. Demographic, socioeconomic, behavioral, and occupational characteristics were assessed using a questionnaire. Actinic cheilitis was evaluated by a clinical examination of the lips, and lesions were classified as grades I (mild), II (moderate), or III (severe). Factors associated with actinic cheilitis were identified by Poisson regression. Fifty-six fishermen were evaluated (mean age = 41.7 years). The average time spent fishing was 27.9 years, with 9.8 hours per day. The majority did not use sunscreen (75%) or lip balm (89.3%) and used uncovered boats (85.7%). Overall, 67.8% had not used dental health services in the last year and 35.7% had their last consultation more than three years ago. The prevalence of actinic cheilitis was 3.5% in grade I, 28.6% in grade II, and 28.6% in grade III. Having white skin and not having visited the dentist in the last three years was associated with a higher prevalence of the lesion while using lip balm was a protective factor. The study showed that riverside fishermen living in a location with a high incidence of ultraviolet radiation had a high prevalence and severity of actinic cheilitis, indicating the need to ensure the use of health services and the adoption of preventive strategies to reduce the occurrence of this potentially malignant oral disorder in these locations.

## Resumo

O estudo avaliou a prevalência de queilite actínica e fatores associados em pescadores residentes em uma comunidade rural ribeirinha na Amazônia, Brasil. Foi realizado um estudo transversal incluindo pescadores artesanais do sexo masculino. Características demográficas, socioeconômicas, comportamentais e ocupacionais foram avaliadas por meio de questionário. A queilite actínica foi avaliada por exame clínico dos lábios, e as lesões foram classificadas em graus I (leve), II (moderado) ou III (grave). Os fatores associados à queilite actínica foram identificados por regressão de Poisson. Foram avaliados 56 pescadores (idade média = 41,7 anos). O tempo médio de pesca foi de 27,9 anos, com 9,8 horas diárias. A maioria não utilizava protetor solar (75%) ou protetor labial (89,3%) e utilizava barcos descobertos (85,7%). Do total, 67,8% não utilizaram serviços de saúde odontológica no último ano e 35,7% realizaram a última consulta há mais de três anos. A prevalência de queilite actínica foi de 3,5% no grau I, 28,6% no grau II e 28,6% no grau III. Cor da pele branca e não ter visitado o dentista nos últimos três anos foram associados à maior prevalência da lesão, enquanto o uso de protetor labial foi fator de proteção. O estudo mostrou que pescadores ribeirinhos residentes em local com alta incidência de radiação ultravioleta apresentaram alta prevalência e gravidade de queilite actínica, indicando a necessidade de garantir a utilização dos serviços de saúde e a adoção de estratégias preventivas para reduzir a ocorrência dessa desordem bucal potencialmente maligna nesses locais.

**Figure:**
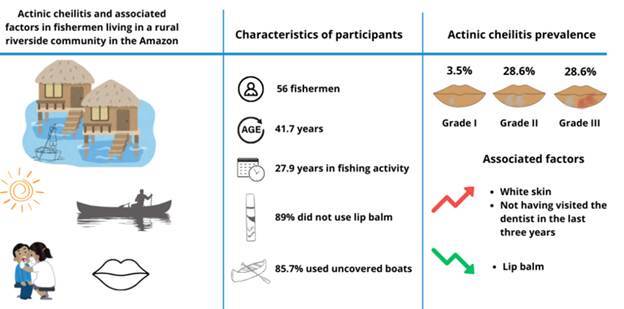

## Introduction

Actinic cheilitis is a degenerative condition of the lip epithelium ^1^. The lesion is considered a potentially malignant disorder, due to the increased risk of developing squamous cell carcinoma (SCC) of the lip ^1^. The malignant transformation rate of actinic cheilitis is estimated at 3.07% ^2^.

The main cause of actinic cheilitis is chronic, prolonged, and excessive exposure to ultraviolet radiation, especially UVB (Ultraviolet B) ^1^. Demographic characteristics such as white skin color, male sex, advanced age; socioeconomic characteristics such as low income and education, occupation with intense sun exposure; and behavioral characteristics including smoking, alcohol abuse, and poor diet are recognized as risk factors. In addition, geographic latitude of residence, genetic predisposition, and immunosuppression have also been identified as factors that contribute to increasing the prevalence or severity of actinic cheilitis ^3^ ^,^ ^4^.

The prevalence of actinic cheilitis may vary among populations ^1^. A systematic review showed an overall prevalence of 2.08% ^5^. However, populations that participate in activities with high sun exposure in Brazil show higher prevalence rates: 34.6% in rural workers ^4^, 18.5% in farmers, 11.6% in construction workers, 9.7% in fishermen, and 7.8% in drivers ^6^.

In Brazil, the North region is marked by the presence of rural riverside communities, that live on or nearby riverbanks. Their way of life and work and subsistence routines are determined by the rhythm of the floods and ebbs of the river waters, and agriculture and artisanal fishing are the predominant activities ^7^. The region is characterized by poor social indicators related to income and education, which are reflected in some of the lowest municipal human development indexes in the country and concentrate on unfavorable health indicators, including oral health ^8^ ^, (^ ^9^. Geographically, as latitude is a determining factor in the amount of radiation on the Earth's surface, the northern states of the country, which are close to the Equator, receive extreme doses of UV radiation (> 11) ^10^.

Considering that the geographic coordinates of the Northern region of Brazil result in a high amount of solar radiation, that fishermen are among the populations with a high prevalence of actinic cheilitis, that artisanal fishing represents one of the most important subsistence activities for the population of this region, as well as for riverside communities in general, and that there are no studies investigating actinic cheilitis among these populations highly exposed to the main cause of this disease, the objective of this study was to evaluate the prevalence of actinic cheilitis in fishermen who live in a rural riverside community in the countryside of Amazonas State, Brazil, as well as to investigate the associated factors.

## Methods

**Figure 1:**
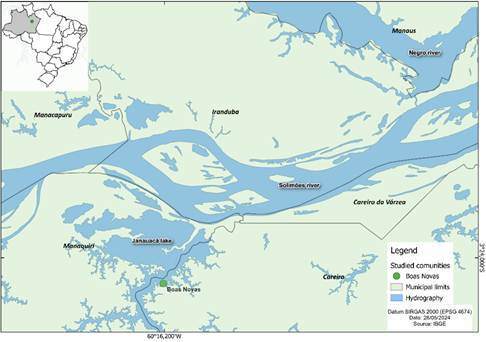
Map indicating the location of the rural riverside community of Boas Novas, municipality of Careiro, Amazonas, Brazil.

A cross-sectional, household-based study was carried out between July and September 2023 in the rural riverside community of Boas Novas. It is a human settlement that represents the traditional communities in the region in terms of its form of organization in fishing production ^7^. This community is located on the shore of Lake Januacá, in the municipality of Careiro, 124 km away from the capital of Amazonas State, Manaus (Figure 1). The majority of the population in Careiro (71.2%) lives in rural areas ^11^ around the lake, in floating houses, or along its banks. The economy is mainly sustained by fishing and agriculture. During the time the study was conducted, the primary care health unit based in the community did not have an oral health team. The fishing activity is predominantly male ^12^. Access to the community was made by speedboat, traveling from the port of Manaus to Boas Novas. Home visits were made on foot or using canoes, depending on where the houses were located.

The study population comprised all adult male fishermen living in the community. According to information from the local fishermen's association, the estimated universe was 70 fishermen. After exhaustive pursuit of all fishermen in the community (up to three attempts), 56 individuals were included in the study representing a statistical power of 87% in a model with three independent variables and a pseudo R^2^ of 0.2, considering a significance level of 0.05.

Data collection was performed by a dentist who was calibrated to assess actinic cheilitis (Kappa = 0.815), and individuals were recruited in their own houses, with up to three attempts. First, an interview was conducted to collect demographic data (sex, age, race/skin color), socioeconomic status (monthly family income and schooling), health-related behaviors (smoking, alcohol consumption, and utilization of oral health services), and occupational features related to actinic cheilitis (cumulative sun exposure in years, daily sun exposure, use of photoprotective measures). Sun exposure was quantified using the following questions: “How many years have you been working in fishing? How many days a week do you work in fishing? How many hours a day do you work in fishing? (Start time / End time)". The questionnaire was developed using the Research Electronic Data Capture (REDCap) platform, which is an open-source application for creating and managing research databases allowing offline access in areas without internet. Next, a clinical examination of the lips was performed under ambient light, following all biosafety protocols.

The outcome was actinic cheilitis. Actinic cheilitis lesions were classified as grades I, II, or III, according to the classification proposed by Medeiros et al. ^13^: Grade I (mild) - dryness, pale areas, white spot; Grade II (moderate) - scaling, lip atrophy, white plaques, white and red spots or plaques, erythema, erosion; Grade III (severe) - blurred demarcation between lip and skin, loss of lip elasticity, fissure, ulceration, crusts, hardened areas, bleeding. The lip examination was conducted at the fishermen's homes, where they remained seated for the inspection, facing natural light. The 16 classification criteria were individually assessed, and each grade was assigned if at least one of the characteristics was observed.

The data were exported from REDCap to Stata SE software, version 15. Initially, the data were analyzed using descriptive statistics. Then, the factors associated with actinic cheilitis were evaluated using Poisson regression analysis, with prevalence ratios and their 95% confidence intervals estimated for the outcome. The variables that had a p-value <0.10 in the bivariate analyses were included in the multiple regression model. The significance level adopted was 0.05.

The consent of the community leader in the Boas Novas community was requested and obtained. The research was submitted to and approved by the Ethics Committee for Research Involving Human Beings of the Federal University of Amazonas (approval number 5.808.323). The fishermen were invited, and those who agreed to participate signed the informed consent.

## Results

**Figure 2:**
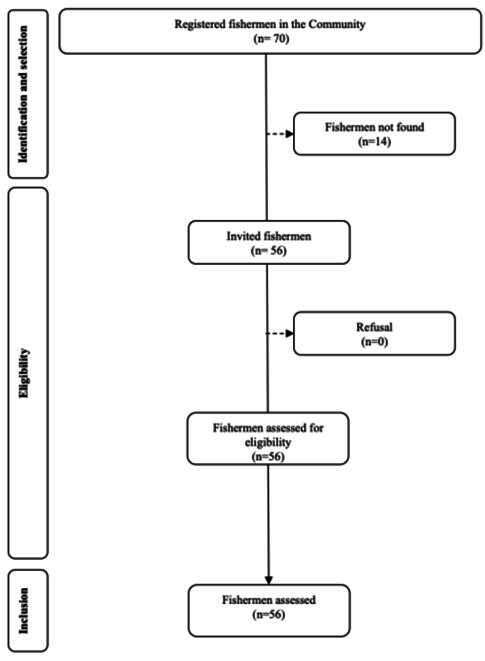
Flow-chart of participants

Fifty-six artisanal fishermen living in the rural riverside community participated in the study (Figure 2). The average age of the fishermen was 41.7 (±13.3) years. Most of them reported having brown skin color/race (53.6%) and an average monthly family income of BRL 1,750.57. The average number of years of schooling was 7.2, and 25% were unable to read or write. Fishing as an exclusive occupation predominated among the participants (64.3%). Among those who had additional employment, 5.4% reported working in agriculture. Table 1 presents the demographic and socioeconomic characteristics of the study participants.

**Table 1:** Demographic and socioeconomic characteristics of participants (n=56).

Variable	Total	Without AC	With AC
Age, mean (SD)	41.7 (13.3)	37.7 (13.1)	44.3 (12.8)
Race/skin color, n (%)			
White	13 (23.2)	2 (9.1)	11 (32.3)
Mixed	30 (53.6)	14 (63.6)	16 (47.1)
Black	13 (23.2)	6 (27.3)	7 (20.6)
Monthly family income, mean (SD)	1,750.57	1,307.73	2,037.12
	(3,924.44)	(536.37)	(5,026.96)
Years of education, mean (SD)	7.2 (4.9)	8.5 (5.7)	6.3 (4.1)
Occupation (n / %)			
Exclusive fishing activity	36 (64.3)	14 (63.6)	22 (64.7)
Fishing and complementary activity	20 (35.7)	8 (36.4)	12 (35.3)

Regarding health-related behaviors, 7.1% of the fishermen reported being smokers, and 39.3% reported consuming alcohol. Regarding the use of oral health services, 67.9% of the fishermen reported not having sought oral health services in the last 12 months and 35.7% of them had their last dental appointment more than three years ago (Table 2).

**Table 2:** Health-related behaviors of fishermen and use of oral health services (n=56).

Variable	Total	Without AC	With AC
Smoking, n (%)			
No	52 (92.9)	21 (95.4)	31 (91.2)
Yes	4 (7.1)	1 (4.6)	3 (8.8)
Number of cigarettes/day (n=4)			
< 1 pack per day	3 (75.0)	1 (100.0)	2 (66.7)
1 pack or more per day	1 (25.0)	0 (0.0)	1 (33.3)
Frequency of alcohol consumption, n (%)			
Never	34 (60.7)	14 (63.6)	20 (58.8)
Once a month or less	7 (12.5)	2 (9.1)	5 (14.7)
2 to 4 times a month	13 (23.2)	4 (18.2)	9 (26.5)
2 to 3 times a week	2 (3.6)	2 (9.1)	0 (0.0)
Most recent dental visit, n (%)			
Up to 1 year	19 (33.9)	9 (40.9)	10 (29.4)
1 year -| 2 years	8 (14.3)	4 (18.2)	4 (11.8)
2 years -| 3 years	9 (16.1)	5 (22.7)	4 (11.8)
> 3 years	20 (35.7)	4 (18.2)	16 (47.0)

Table 3 presents the occupational features. The average time spent fishing was 27.91 years (± 12.99 SD). Just over half (53.6%) worked seven days a week, with an average of 9.86 hours per day (± 2.86 SD). Most of them did not use sunscreen (75%) or lip balm (89.3%) and operated boats or canoes without sun protection (85.7%). On the other hand, all wore hats or caps and 98.21% wore long-sleeved shirts.

**Table 3:** Occupational characteristics of riverine fishermen (n=56).

Variable	Total	Without AC	With AC
Years in fishing activity, mean (SD)	27.9 (13.0)	26.2 (13.9)	29.0 (12.5)
Weekly frequency of fishing, n (%)			
2 to 4 times	15 (26.8)	8 (36.4)	7 (20.6)
5 to 6 times	11 (19.6)	6 (27.2)	5 (14.7)
7 times	30 (53.6)	8 (36.4)	22 (64.7)
Hours per day in fishing activity, mean (SD)	9.9 (2.9)	9.0 (3.2)	10.4 (2.5)
Use of photoprotective measures, n (%)			
Sunscreen			
Yes	14 (25.0)	8 (36.4)	6 (17.7)
No	42 (75.0)	14 (63.6)	28 (82.4)
Lip balm			
Yes	6 (10.7)	1 (4.6)	5 (14.7)
No	50 (89.3)	21 (95.4)	29 (85.3)
Hat or cap			
Yes	56 (100.0)	22 (100.0)	34 (100.0)
Long-sleeved shirt			
Yes	55 (98.2)	22 (100.0)	33 (97.1)
No	1 (1.8)	0 (0.0)	1 (2.9)
Boat with cover, n (%)			
Yes	8 (14.3)	3 (13.6)	5 (14.7)
No	48 (85.7)	19 (86.4)	29 (85.3)

SD = standard deviation

Clinical characteristics of actinic cheilitis were observed in 60.7% of the fishermen. Moderate actinic cheilitis were observed in 28.6% of the fishermen and the same proportion had severe cheilitis. Mild cheilitis was present in 3.5% of the fishermen. Among the 34 individuals affected by actinic cheilitis, white and red spots or plaques were the most prominent characteristics, observed in 79.4% of the fishermen (93.75% in Grade II; 75% in Grade III). Blurred demarcation between the lip and skin, is present in 47% of them (100% in Grade III).

Regression analyses revealed that having white skin (PR=1.60; 95%CI 1.10-2.34) and not having visited a dentist in the last three years (PR=1.68; 95%CI 1.13-2.50) were associated with a higher prevalence of actinic cheilitis while using lip sunscreen (PR=0.59; 95%CI 0.38-0.91) was a protective factor (Table 4).

**Table 4:** Association between actinic cheilitis and socioeconomic characteristics, healthrelated behaviors, use of oral health services and occupational characteristics of riverine fishermen

Variable	PR_crude_ (95%CI)	p-value	PR_adjusted_ (95%CI)	p-value
Age	1.02 (1.00-1.03)	0.052^a^		
Skin color (ref.: black/brown)				
White	1.58 (1.10-2.28)	0.014^*^	1.60 (1.10-2.34)	0.015^*^
Household income (R$1,000.00)	1.02 (1.00-1.03) ^*^	0.006^*^		
Can read/write (ref.: no)				
Yes	1.08 (0.65-1.82)	0.761		
Years of study with approval	0.96 (0.92-1.01)	0.121		
Number of people per room	1.03 (0.70-1.51)	0.898		
Smoking (ref.: no)				
Yes	1.26 (0.68-2.32)	0.464		
Alcohol consumption (ref.: no)				
Yes	1.08 (0.71-1.66)	0.718		
Years in fishing activity	1.01 (0.99-1.02)	0.449		
Hours per week spent fishing	1.01 (1.00-1.02)^a a^	0.071		
Dental appointment (ref.: ≤3 years)				
> 3 years	1.60 (1.08-2.40)	0.020^*^	1.68 (1.13-2.50)*	0.011^*^
Use of sunscreen (ref.: no)				
Yes	1.56 (0.81-2.97)	0.181		
Use of lip balm (ref.: no)				
Yes	0.70 (0.45-1.07)	0.100^a^	0.59 (0.38-0.91)*	0.018^*^
Boat with cover (ref.: no)				
Yes	0.97 (0.54-1.74)	0.910		

## Discussion

The study findings showed a high prevalence of actinic cheilitis, especially in its more severe stages. Having white skin and not having utilized oral health services for more than three years was associated with a higher prevalence of actinic cheilitis. Using lip balm, although infrequent in the study population, was associated with a lower prevalence of the outcome.

Actinic cheilitis, in varying degrees, was observed in approximately 60% of the fishermen, an extremely high prevalence than the global average of 2% ^5^, but also considerably higher than that of other populations with occupational sun exposure, both in Brazil and worldwide, which range from 7.8% ^6^ to 34% ^4^. The prevalence of actinic cheilitis is influenced by multiple factors. For instance, other Brazilian states also located in areas of high UV radiation, such as Sergipe, reported lower prevalence rates (11.4%) ^14^. On the other hand, regions with lower UV radiation intensity, such as Spain, have shown higher prevalence rates (31.3%) ^15^. The studied population is exposed to intense sun exposure, characteristic of fishing occupation, combined with the geographical latitude of their place of residence and work, which contributes to a higher risk of actinic cheilitis (3, 15). Furthermore, this population has been fishing for many years (an average of 28 years, ranging from 14 to 40 years), and nearly three-quarters of them fish between 5 and 7 days per week, and approximately 10 hours per day on average, which means high exposure in both duration and intensity to the primary etiological agent of the disorder ^16^. These occupational characteristics are similar to those of rural workers in other regions of Brazil ^17^. Actinic cheilitis lesions develop slowly as a result of chronic and prolonged sun exposure (1, 2). There is evidence that individuals with at least 14 years of chronic sun exposure already exhibit actinic cheilitis lesions on the lips ^18^. In Amazonas, the UV index can reach levels considered "very high" to "extreme" throughout the year ^10^.

White skin color increased the prevalence of the lesion by 60%. This result is coherent and consistent with the literature ^4^. The biological plausibility is the lower concentration of melanin, a pigment with a protective effect against UV radiation, in the basal layer of keratinocytes in white-skinned individuals ^16^. It is interesting to note that, among the fishermen evaluated, only about a quarter self-identified as white, while almost 60% identified as brown, which characterizes the population of the northern region of Brazil well ^19^. In other studies conducted in Brazil, white skin color predominated ^4^ ^,^ ^20^.

Fishermen whose last dental appointment was more than three years ago showed a higher frequency of actinic cheilitis. The association between these variables has been little studied. However, it is relevant, especially due to the high proportion of just over one-third of fishermen who did not use oral health services during this period. The absence of an oral health team at the primary care health unit in the community can contribute to this result. To have a dental appointment, the fisherman would have to travel to the urban area of ​​the municipality, which means both financial and time-related expenses. Besides, the high daily and weekly workload of fishermen itself constitutes an organizational barrier to accessing health services, even if the oral health team was present at the primary healthcare unit. This should be taken into account when planning healthcare services in these locations. Low utilization of oral health services is common among riverine communities. Another study conducted in rural riverside communities in the Amazon showed that one-quarter of individuals had not used oral health services in the last three years or had never used them ^21^. Regular use of oral health services is protective for other oral health outcomes, such as tooth loss in rural riverside communities ^22^ and caries in Brazilian adults and elderly individuals in urban areas ^((^ ^23^ ^, (^ ^24^. This finding shows the importance of both access to and utilization of oral health services in preventing or early diagnosing actinic cheilitis and, therefore, the need to overcome the geographic, financial, and organizational barriers to accessing health services that characterize the Amazon region ^22^.

Actinic cheilitis lesions have a slow and asymptomatic progression ^1^, and dental consultations may represent an opportunity for early detection and also for educational actions to prevent the disease. Early detection can minimize the progression of invasive squamous cell carcinoma ^3^ through the prescription of protective measures, such as sunscreen ^25^. Therefore, in addition to the availability of dental services, it is necessary to ensure that professionals can diagnose early lesions, since the diagnosis may be difficult in the early stages ^25^. The literature already highlights the need for ongoing health education to ensure that dentists at the primary healthcare level within the public health system can perform proper oral clinical examinations to detect potentially malignant disorders or malignant lesions. It would help reduce of barriers associated with the early diagnosis. It is also necessary to surpass the traditional model of exclusively focusing on the clinical aspects of lesions, by adopting educational and health-promoting actions, which may involve other professionals in the health team, and not just the dentist ^26^.

In the present study, the use of lip balm was a protective factor against the occurrence of actinic cheilitis, with a 40% lower prevalence among those who had their lips protected. This photoprotective measure was not widely adopted by the studied population and is also not usually reported by other fishermen and rural workers ^14^ ^,^ ^17^. Although the use of lip balm was associated with a lower occurrence of actinic cheilitis in the fishermen in this study as well as in other fishermen ^14^, a systematic review did not identify the measure as a protective factor ^16^. Photoprotection may require the combination of more than one measure. Most rural workers use hats or caps as a protective measure ^17^, as observed in the present study, probably because they are the most accessible ones. Nonetheless, the hat alone may not be effective in protecting against actinic cheilitis. In most cases, hats and caps are not made of materials with effective photoprotection, and the lips are not fully protected by their shade ^18^. It is necessary to recognize that lip balm is expensive and may not be accessible to a large portion of the population, especially in rural populations, which are generally characterized by unfavorable socioeconomic conditions ^4^, just like the population in the present study, in which a quarter of the fishermen were illiterate and had a monthly household income ranging from one and two minimum wages.

In this study, socioeconomic status and other health-related behaviors, such as smoking and alcohol consumption, were not associated with the occurrence of actinic cheilitis. The literature suggests that these factors may contribute to an increase in the frequency or severity of the lesion (3, 4). It is important to note, however, that the studied community is relatively homogeneous in terms of socioeconomic characteristics. This may hinder the identification of an association between these factors and cheilitis, as there is little variation among them.

Some limitations of the study should be pointed out. The cross-sectional design does not allow for assuming temporality between the events, especially between exposures such as the use of protective measures and the utilization of oral health services, and the outcome. Skin color is an immutable characteristic, which allows for establishing a more reliable causal association. The number of participants may also represent a limitation. Although the study sought to include the universe of fishermen living in the community, the statistical power of the study may be limited to identifying existing associations.

The results allow us to conclude that the prevalence of actinic cheilitis in fishermen living in a rural riverside community in the interior of the state of Amazonas was high, especially in its moderate and severe stages. White skin color and use of oral health services for more than three years previously were associated with a higher prevalence of the lesion. On the other hand, the use of lip balm was associated with a lower prevalence of the condition. As far as the authors are aware, the study is pioneering for the population under investigation. The high prevalence of actinic cheilitis lesions among Amazonian riverside fishermen residing in areas with high ultraviolet exposure is alarming. Therefore, more comprehensive studies involving communities with similar geographic, and socioeconomic characteristics and lifestyles are needed. Furthermore, it is imperative to ensure universal and comprehensive access to oral health services in these regions, considering that early diagnosis reduces patient morbidity. In addition, lesion progression can be halted in its early stages, and malignant transformation can be minimized by adopting protective measures, which highlights the importance of dentists in the control and treatment of the disorder. At the same time, acting preventively, oral health services should also plan and implement educational strategies related to photoprotective measures that engage the community and ensure access to them, so the population can effectively benefit from the use of oral health services for the control of actinic cheilitis. Thus, the evaluation of oral health services and their strategies for the prevention of disorders such as actinic cheilitis is also important and necessary.
